# Genetic Diversity Characterization of Porcine Reproductive and Respiratory Syndrome Virus Isolates in Romania, Based on Phylogenetic Analysis

**DOI:** 10.3390/ijms130912046

**Published:** 2012-09-21

**Authors:** Mihaela Zaulet, Maria Rodica Gurau, Vlad Petrovan, Laura Buburuzan

**Affiliations:** 1 Department of Biochemistry and Molecular Biology, University of Bucharest, 91–95 Splaiul Independentei, 5th district Bucharest, Romania; E-Mails: zaulet_mihaela@yahoo.com (M.Z.); petrovan.vlad@gmail.com (V.P.); 2 Faculty of Veterinary Medicine, University of Agronomical Science and Veterinary Medicine Bucharest, 105 Splaiul Independentei, 5th district Bucharest, Romania; E-Mail: otelea_maria@yahoo.com

**Keywords:** porcine reproductive and respiratory virus, *ORF5*, *ORF7*, swine, phylogeny

## Abstract

Porcine reproductive and respiratory syndrome (PRRS) is a disease produced by the (PRRS) virus, characterized by endemic evolution in the majority of countries, which remains in actuality being a permanent threat to health and economic free farms, as well as for those infected. The aim of this study was to evaluate the genetic diversity of Romanian PRRSV isolates from the four most important pig farms in Romania by comparing the nucleotide sequences obtained for *ORF5* and *ORF7* with a wide range of sequences from GenBank belonging to the main types of PRRSV; the type 1. Eighteen different sequences were obtained for *ORF5* gene and 10 for *ORF7* gene. One Romanian isolate (Rom3) was found in three of the four different investigated farms. The phylogenetic analysis revealed that the Romanian PRRSV nucleotide sequences clustered in three groups within the subtype 1 of the virus. The analysis of amino acid sequences evidenced for GP5 and N-nucleocapsid proteins confirmed that the Romanian virus belonged to type 1.

## 1. Introduction

Porcine reproductive and respiratory syndrome (PRRS) is the pathological dominant of the 90 decade for pigs. PRRS is a viral disease, characterized by endemic evolution in the majority of countries, which remains in actuality being a permanent threat to health and economic free farms, as well as for those infected.

In March 1991, a group of researchers from the Institute of Lelystad, Netherlands, was able to isolate and identify the etiologic agent and experimental reproduction of the disease [1,2]. It has been established that PRRS is caused by a virus that belongs to a new group of RNA viruses. Genome organization, structure and biology are similar to those of increased lactate dehydrogenase virus (dairy Dehydrogenase Elevating virus LDV), virus infectious equine arteritis (equine arteritis virus EAV) and the Ebola virus simian apes (simian hemorrhagic fever SHFV) [3]. Comparison of PRRS virus, lactate dehydrogenase (LD) and equine arthritis (AE), allows for the possibility of a common phylogenetic tree, in which the PRRS virus is closer to the LD virus than the AE virus. Hence, the hypothesis that PRRS virus is a variant of LD virus adapted to pigs.

Porcine reproductive and respiratory syndrome virus belongs to the family *Arteriviridae* in the order of *Nidovirales* [4]. It has dimensions of 45–70 nm, icosahedron symmetry; its envelope covers an internal spherical nucleocapsid of 25–30 nm [5]. Genetic material is represented by a simple linear RNA molecule (15,000 nucleotide, with positive polarity). The viral genome consists of nine open reading frames (ORF): *ORF1a*, *ORF1b*, *ORF2a*, *ORF2b*, *ORF3*, *ORF4*, *ORF5*, *ORF6*, *ORF7* [6,7].

PRRSV is divided into two genotypes: type 1 and type 2. Comparison of antigenic different strains demonstrated that European and American strains are approximately 60% similar to each other [8].

The purpose of the present study was to investigate the PRRSV genetic variability based on sequence transcription, amplification and sequencing of genes of *ORF7* and *ORF5*, using different tissues homogenates, collected from four pig farms in Romania. To confirm the occurrence of type 1 of PRRSV in Romania, both genes of *ORF5* and *ORF7* were sequenced.

The studied PRRS outbreaks have appeared in four important counties in different geographical areas in Romania.

## 2. Results and Discussion

**Figure 1:**
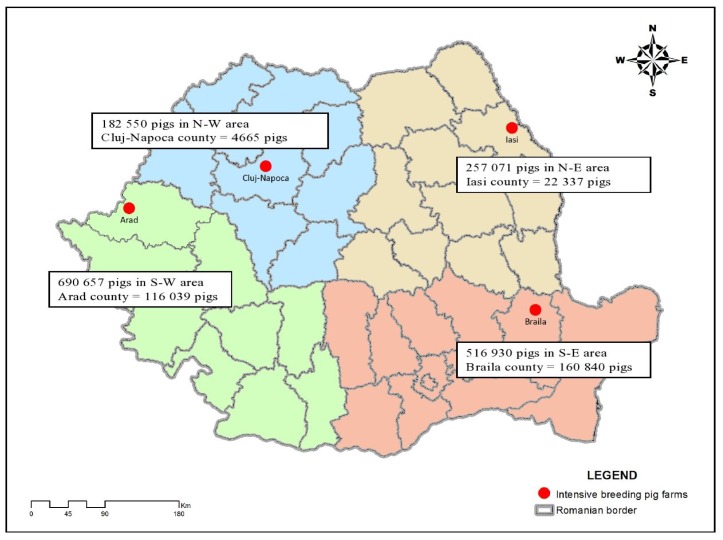
Political map of Romania showing the number of pigs from different areas.

In order to investigate the PRRSV genetic variability in four pig farms in Romania, 605 samples were collected from different individuals. From the total amount of samples, 33 were positive for PRRSV by PCR. We were able to conduct the investigations on both *ORF5* and *ORF7* genes on 23 samples but for the other 10 samples due to the small quantity of initial tissue, we were only able to fulfill the investigations on the *ORF5* gene (Table 1). In our study, we divided Romania into four different areas (Figure 1).

In Table 2, we described the areas, the total number of swine and the number of samples collected and analyzed from four geographical areas.

The *ORF5* and *ORF7* specific PCR products were sequenced to confirm the occurrence of type 1 of PRRSV in Romania. The obtained nucleotide sequences were aligned using CLUSTAL W program [9], resulting in a 606 nucleotides alignment for *ORF5* gene and a 387 nucleotides alignment for *ORF7*. In our study, we indentified 18 sequences for *ORF5* and 10 sequences for *ORF7* with at least one nucleotide point mutation compared with data already available in the GenBank.

Interestingly, based on *ORF5* gene analysis, among the 18 different Romanian isolates, Rom3 PRRSV strain was detected in three of the four pig farms investigated (Braila, Iasi, Arad counties), while Rom5 PRRSV strain was found in 2 different pig farms (Braila and Arad counties). Taking into account the number of identical nucleotide sequences obtained for gene *ORF5*, PRRSV strain Rom3 was isolated from 14 different individuals (10 pigs from Braila farm, three pigs from Iasi farm and one from Arad farm). Based on the analysis of *ORF7* nucleotide sequence, the new PRRSV strains are different among all Romanian pig farms investigated, but the Rom4 isolate is the most prevalent in Braila farm (found in ten different individuals, Table 1).

**Figure 2:**
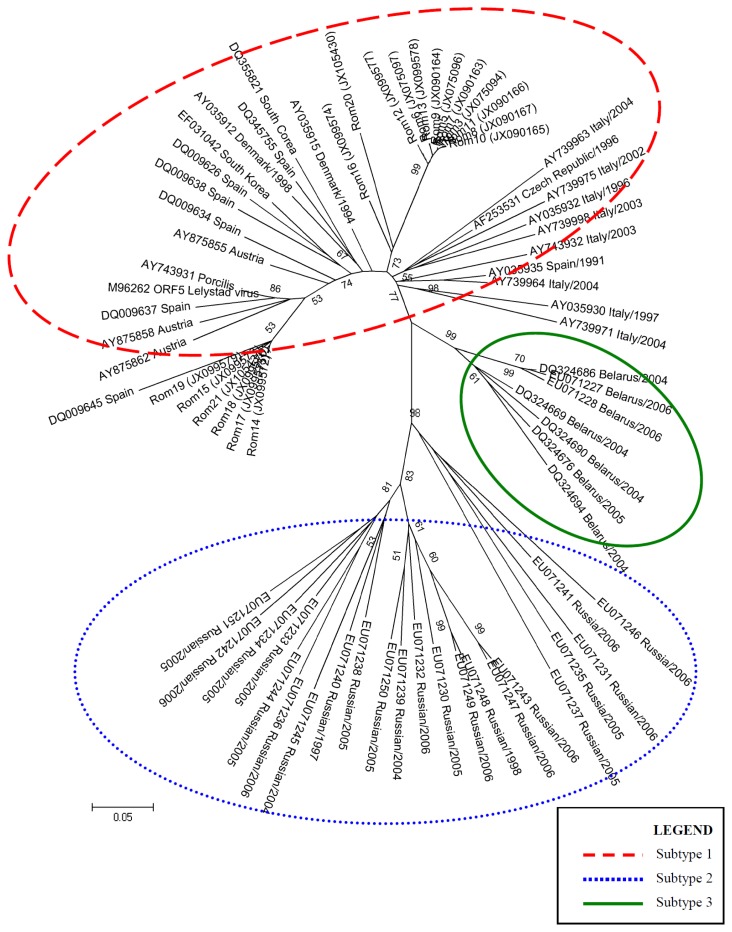
Maximum Likelihood tree based on *ORF5* for the Romanian sequences, together with similar sequences from GenBank Database. The evolutionary distances were computed using the Tamura-Nei + G + I method. The bootstrap values adjacent to the main nodes represent the probabilities based on 1000 replicates.

In 2008, Stadejek *et al.* proposed the division of the type 1 PRRSV genotype into three subtypes, based on *ORF7* analysis [10]. Two phylogenetic trees were constructed, based on the complete sequences obtained for *ORF5* gene and *ORF7* gene of PRRSV Romanian isolates, together with a wide range of sequences selected from GenBank (Table 3). The evolutionary history was inferred using the Maximum Likelihood method [11]. The bootstrap consensus tree inferred from 1000 replicates is taken to represent the evolutionary history of the taxa analyzed [12]. Branches corresponding to partitions reproduced in less than 50% bootstrap replicates are collapsed. The tree is drawn to scale, with branch lengths in the same units as those of the evolutionary distances used to infer the phylogenetic tree. The evolutionary distances were computed using the Tamura-Nei method [13] and are in the units of the number of base substitutions per site. The rate variation among sites was modeled with a gamma distribution (shape parameter = 1) with Invariant. The differences in the composition bias among sequences were considered in evolutionary comparisons [14]. The analysis involved 72 nucleotide sequences for *ORF5* and 99 nulcleotide sequences for *ORF7*. Codon positions included were 1st + 2nd + 3rd + Noncoding. All ambiguous positions were removed for each sequence pair. Evolutionary analyses were conducted in MEGA4 [15] (Figures 1 and 2). The type 1 PRRSV clade is clearly divided into three clusters corresponding to subtypes 1, 2 and 3 [10].

The phylogenetic tree constructed, based on *ORF5* gene (Figure 2), shows that all Rom isolates cluster within the type 1 PRRSV strain, indicating a high similarity with other virus strains belonging to the subtype 1.

In our phylogeny study on *ORF5* gene, we used Maximum Likelihood method with gamma distribution. In the analysis of the phylogenetic tree, we identified three major clades according with the three subtypes indicated by Stadejek *et al.* [10]. The isolates from Russia are included in subtype 2, from Belarus are included in subtype 3, and Romanian sequences were distributed in a monofiletic group according to subtype 1 from Type 1. In this group, our Romanian sequences: Rom11, 10, 8, 3, 7, 5, 9, 13, 6, 12, 16 and 20 form a distinct clade. The Romanian sequences Rom15, 21, 18, 17, 14 and 19 form a clade with the sequences from Spain, Austria, Lelystad virus and Porcilis vaccine.

The results from our study are in concordance with the analysis made by Stadejek *et al*. in 2008 [10] and permit us to include the Romanian isolates in subtype 1 on PRRSV.

**Figure 3:**
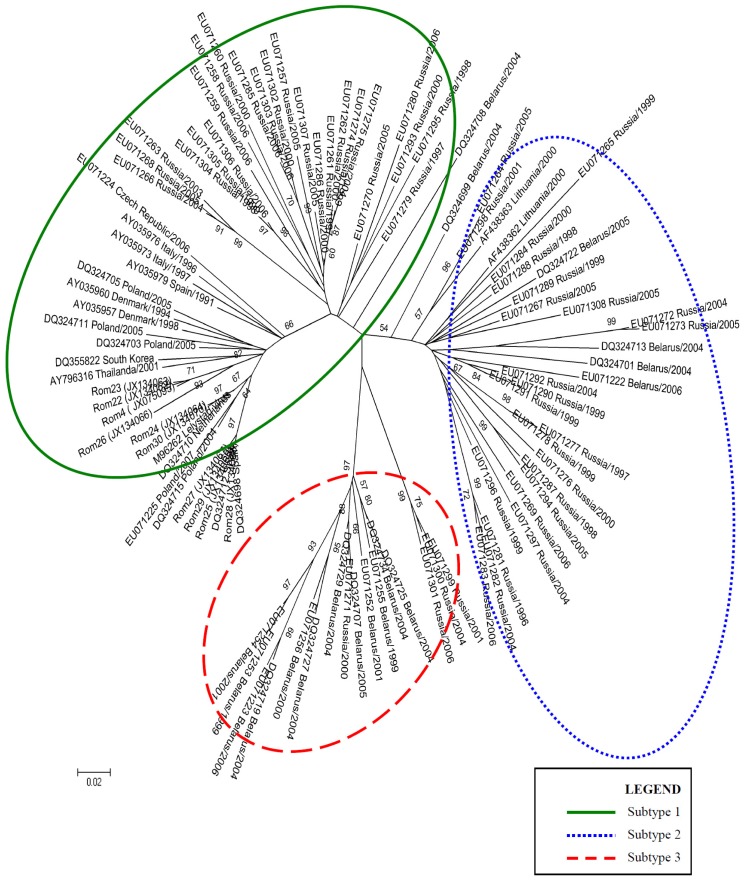
Maximum Likelihood tree based on *ORF7* for the Romanian sequences, together with similar sequences from GenBank Database. The evolutionary distances were computed using the Tamura-Nei + G + I method. The bootstrap values adjacent to the main nodes represent the probabilities based on 1000 replicates.

The phylogenetic tree obtained based on *ORF7* sequences (Figure 3) shows the same topology as the tree based on *ORF5* sequences. The division of the type 1 clade into three large subtypes 1, 2 and 3, isolates is also noticeable as suggested by other authors as well [10]. We realized that one sequence, DQ324708, is not included in the three subtypes described; all other sequences were included in the three subtypes.

The results obtained for *ORF7* Romanian isolates are in accordance with those obtained for *ORF5* isolates. In the case of Romanian sequences, were evidenced two monophyletic groups. One distinct monophyletic group constituted of sequences Rom26, 4, 22 and 23; the second monophyletic group were represented by the *ORF7* sequences of Porcilis vaccine (DQ324710), Lelystad virus (M96262) and sequences from Spain (DQ324698 and DQ324712).

**Figure 4:**
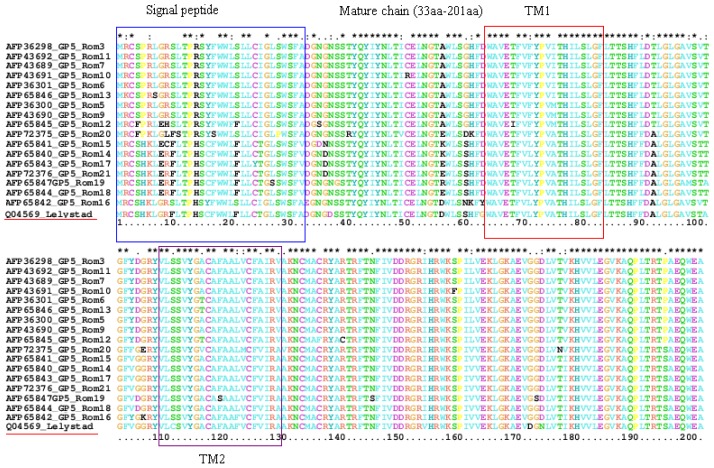
The amino acid sequences of GP5 (201 amino acids) divided into signal peptide (1st aa to 32nd aa) and the mature chain (33rd aa to 201st aa). The mature chain contains two transmembrane elements (TM1—64th aa to 84th aa, TM2—109th aa to 129th aa).

**Figure 5:**
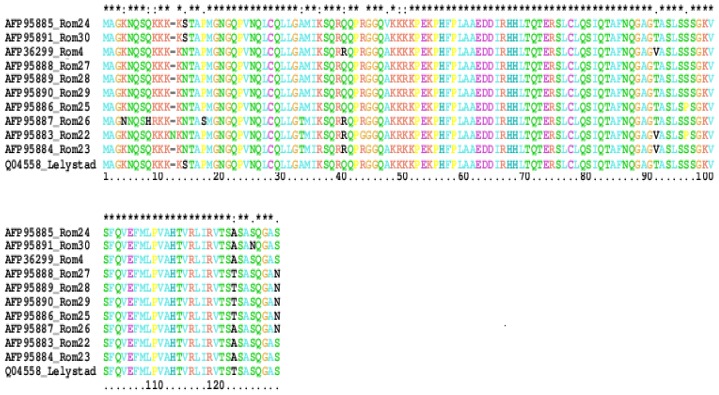
Alignment of the deduced amino acid sequences of ORF7 protein of 10 Romanian isolates and Lelystad virus (M 96262). Substitutions are indicated by the amino acid letter codes.

The deduced amino acid sequences encoded by both *ORF5* and *ORF7* genes were aligned using CLUSTAL W program [9], resulting in a 201 amino acids alignment, corresponding to *ORF5* gene (Figure 4) and a 128 amino acid alignment corresponding to *ORF7* gene (Figure 5). A single exception can be noticed: the amino acid sequence for Rom22 isolate from Braila farm has an asparagine inserted at position 12 of the sequence.

It is known that GP5 protein is very polymorphic [16,17] being under the permanent pressure of selection forces due to its exposed position at the exterior of the virion [18]. This is why GP5 is highly informative regarding the evolution and origin of different PRRSV isolates. In particular, some known functional domains of GP5, such as the signal peptide, mature chain with transmembrane regions, some motifs in GP5 like primary neutralizing epitope (PNE) and decoy epitope were also analyzed according to a previous report [19]. Our aim was to investigate the amino acid difference among the subtype 1 of Romanian isolates. The GP5 amino acid sequences of 17 PRRSV isolates were aligned, together with the Lelystad virus sequence. Multiple alignments of GP5 sequences of Romanian PRRSV isolates indicated that all 17 isolates encode a GP5 protein of 201 amino acid residues (Figure 4).

Nucleocapsid protein (N) is encoded by *ORF7* gene [18,20] and has 128 amino acids in type 1 PRRSV. The *N*-terminus of the protein interacts with the viral genomic RNA [21,22] and the *C*-terminus has the role of maintaining the tertiary structure of N protein [23]. The deduced amino acid sequences for all 10 different Romanian isolates aligned with CLUSTAL W program [21] reveal no extended hypervariable regions, as expected due to the fact that nucleocapsid protein is a very conserved molecule.

The distribution of sequence diversity across the ORF7 protein was investigated for all 10 sequences analyzed in this study. The analysis sequences which contains 128 amino acids demonstrate that Romanian isolates have some amino acid substitutions compared with Lelystad virus: Rom22 isolate with an asparagine inserted between positions 11 and 12 of the alignment, and one substitution in position 42, Rom26 with three amino acid substitutions in positions 4, 8 and 16 and Rom30 with one amino acid substitution in position 124 (Figure 5).

## 3. Experimental Section

### 3.1. Sampling and RNA Extraction

The biological samples were supplied from four pig farms contaminated with PRRSV, with clinical signs of disease. The pig farms were from different geographical areas of Romania: Braila, Arad, Cluj-Napoca and Iasi counties.

Samples used in this study were collected post mortem from all selected pigs from 2010 to 2012. Also, all the samples were collected as early as possible after exitus, deposited in RNA, later buffer, for transportation, and then stored at −80 °C.

Numbers of samples totaled 605 were collected: 260 from pigs from Braila farm, 85 from pigs from Iasi farm, 200 from pigs belonging to Arad farm and 60 from pigs from Cluj-Napoca farm. The tissues used for PRRSV-RNA extraction were tonsil, lung, mediastinal lymph node, liver, spleen, and kidney.

Total RNA was extracted using the RNeasy Mini Kit (Qiagen, Austin, TX, USA). 200μL tissue homogenate was processed in accordance with the manufacturer’s instructions.

### 3.2. PCR Analysis

Two genes specific to PRRSV were analyzed in our study: *ORF7* (open reading frame 7) and *ORF5* (open reading frame 5).

For *ORF7* gene, One-Step RT-PCR was carried out in a final volume of 25 μL (23, 5 μL PCR mix and 1, 5 μL RNA extract). The PCR mix contains 40 U/μL RNasin (Promega, Madison, WI 53711, USA) (0, 1 μL), 5 μM forward primer, 5 μM reverse primer, and 1 μL One-Step RT-PCR Enzyme mix (Qiagen), dNTPs, Buffer 5X (Qiagen), and nuclease-free water. The following primers were used for *ORF7* One-Step RT-PCR: *ORF7* B-forward primer (5′-GCCCCTGCCCAICACG-3′), (TibMolBiol, Berlin, Germany) and *ORF7* C-reverse primer (5′-TCGCCCTAATTGAATAGGTGA-3′), (TibMolBiol, Berlin, Germany). These primers are used for the diagnosis of PRRSV European-type strains in accordance with the Lelystad virus sequence.

PCR was performed using a iCycler-BIO-RAD thermocycler, with the following program: 30 min at 50 °C, 1 cycle; 15 min at 95 °C, 1 cycle; 45 s at 95 °C, 45 s at 55 °C, 60 s at 72 °C, 45 cycles; and 4 °C ∞.

The complete amplification of *ORF5* gene sequence was accomplished by nested RT-PCR. The first step PCR was set up in a volume of 25 μL (23, 5 μL PCR mix and 1, 5 μL RNA extract). The PCR mix and the thermocycler program (iCycler-BIO-RAD) were the same as for *ORF7* PCR. The following primers were used for *ORF5* first step PCR: EU-5F-forward primer (5′-TGATCA CATTCGGTTGCT-3′), (TibMolBiol, Berlin, Germany) and EU-5R-reverse primer (5′-GGGCGT ATATCATTATAGGTG-3′) (TibMolBiol, Berlin, Germany).

The second step PCR was set up in a volume of 25, 15 μL (23, 65 μL PCR mix and 1, 5 μL DNA). The PCR mix contains 5 μM forward primer, 5 μM reverse primer, 0, 15 μL OneStep RT-PCR Enzyme mix (Qiagen) and 4 μL MgCl_2_ (25 M), dNTPs, Buffer5X and nuclease-free water. The following primers were used for *ORF5* second step PCR: EU5B-forward primer (5′-CAA TGAGGTGGGCIACAACC-3′), (TibMolBiol, Berlin, Germany) and EU5C-reverse primer (5′-TAT GTIATGCTAAAGGCTAGCAC-3′) (TibMolBiol, Berlin, Germany). PCR was performed using a iCycler-BIO-RAD thermocycler, with the following program: 5 min at 95 °C, 1 cycle; 45 s at 95 °C, 45 s at 55 °C, 60 s at 72 °C, 45 cycles; 72 °C at 10 min, 1 cycle and 4 °C ∞.

The PCR products were visualized on 2% agarose gels stained with ethidium bromide.

### 3.3. Sequencing and Phylogenetic Analysis

The PCR reaction products were purified using Wizard^®^ PCR Preps DNA Purification System (Promega, Madison, WI, USA), and the concentration and purity of the products were evaluated by spectrophotometry (Eppendorf BioPhotometer, Hamburg, Germany). The DNA sequencing reactions were performed for both forward and reverse strands using BigDye Terminator Kit v3.1 (Applied Biosystems, Foster City, CA, USA).

The sequencing was performed on a 3130 Genetic Analyzer (Applied Biosystems, Foster City, CA, USA). The sequences obtained were proofread manually, truncated to the real dimensions of the genes (606 bp for *ORF5* gene and 387 bp for *ORF7* gene) using BioEdit version 7.1.3.0 [24] and aligned using CLUSTAL W application from MegAlign program (DNASTAR, Intelligenetics, Madison, WI, Wisconsin). *ORF5* and *ORF7* corresponding sequences were compared with a set of reference sequences selected from GenBank to cover a wide range of genetic and geographic diversity from Europe. All the Romanian isolates received GenBank accession numbers (Table 1). The set of reference sequences used to construct the phylogenetic tree is presented in Table 2. The two phylogenetic trees were generated from the aligned sequences in MEGA4 program [25] using a Maximum Likelihood method [15]. The evolutionary distances were computed using the Tamura-Nei + gamma + I model [26] and are in the units of the number of base substitutions per site. Percentage reliability values at each internal node of the trees were obtained by performing 1000 bootstrap analyses.

## 4. Conclusions

This is the first extensive study on Romanian PRRSV isolates that provides information about the genetic diversity of this virus in the four most important pig farms in Romania. This study completes our early data on the first two Romanian strains analyzed [27]. The results obtained from the phylogenetic trees together with the pairwise nucleotide sequence identity confirm the affiliation of all Romanian isolates to the subtype 1 of the virus. The evolution of PRRSV in the Romanian pig farms follows four distinct directions.

For *ORF5*, the isolates from Romanian sequences were distributed in a monofiletic group according to subtype 1 from Type 1. A distinct clade is seen for the Romanian sequences Rom11, 10, 8, 3, 7, 5, 9, 13, 6, 12, 16 and 20, while the Romanian Rom15, 21, 18, 17, 14 and 19 are grouped along with sequences from Spain, Austria, Lelystad virus and Porcilis vaccine.

In the case of *ORF7*, we observed the existence of two monophyletic groups. The Romanian sequences Rom 26, 4, 22 and 23 belong to a different monophyletic group compared to sequences of Porcilis vaccine (DQ324710), Lelystad virus (M96262) and sequences from Spain (DQ324698 and DQ324712).

For the amino acid sequences for ORF5, we identified two hypervariable regions, one in the signal peptide and one in the beginning of the mature chain. Regarding N protein, Romanian isolates have some amino acid substitutions compared with Lelystad virus: Rom22 isolate with an asparagine inserted between position 11 and 12 of the alignment, and one substitution in position 42, Rom26 with three amino acid substitutions in positions 4, 8 and 16, Rom30 with one amino acid substitution in position 124.

## Figures and Tables

**Table 1:** Romanian PRRSV isolates and their GenBank accession numbers used in this study.

Location (City)	Sample identification number	*ORF5*		*ORF7*	

		Isolate	GenBank accession No.	Isolate	GenBank accesion No.
Braila	1069	Rom6	JX075097	Rom4	JX075095
Braila	1070	Rom3	JX075094	Rom4	JX075095
Braila	1071	Rom3	JX075094	Rom4	JX075095
Braila	1076	Rom3	JX075094	Rom4	JX075095
Braila	1077	Rom3	JX075094	Rom4	JX075095
Braila	1078	Rom3	JX075094	Rom4	JX075095
Braila	1079	Rom5	JX075096	Rom4	JX075095
Braila	1104	Rom5	JX075096	Rom4	JX075095
Braila	1105	Rom3	JX075094	Rom4	JX075095
Braila	1106	Rom3	JX075094	Rom4	JX075095
Braila	776	Rom12	JX099577	Rom22	JX134062
Braila	865	Rom3	JX075094	Rom23	JX134063
Braila	849	Rom3	JX075094	Rom4	JX075095
Braila	836	Rom3	JX075094	Rom4	JX075095
Braila	835	Rom13	JX099578	Rom23	JX134063
Iasi	1	Rom3	JX075094	-	-
Iasi	4	Rom7	JX090163	-	-
Iasi	6	Rom8	JX090167	-	-
Iasi	7	Rom3	JX075094	-	-
Iasi	8	Rom3	JX075094	-	-
Iasi	9	Rom9	JX090164	-	-
Cluj-Napoca	70	Rom21	JX105431	Rom 30	JX134070
Cluj-Napoca	73	Rom14	JX099572	Rom24	JX134064
Cluj-Napoca	74	Rom18	JX099576	Rom28	JX134068
Cluj-Napoca	75	Rom17	JX099575	Rom27	JX134067
Cluj-Napoca	77	Rom20	JX105430	Rom 30	JX134070
Cluj-Napoca	79	Rom15	JX099573	Rom25	JX134065
Cluj-Napoca	82	Rom19	JX099579	Rom29	JX134069
Cluj-Napoca	87	Rom16	JX099574	Rom26	JX134066
Arad	13	Rom5	JX075096	-	-
Arad	14	Rom10	JX090165	-	-
Arad	18	Rom3	JX075094	-	-
Arad	19	Rom11	JX090166	-	-

**Table 2:** Total number of swine from the geographical areas and the number of swine in the intensive breeding farms.

Geographical Area	Total number of swine	Total number of swine in county	Number of samples analyzed
N-E	257,071	22,337/Iasi	85
S-E	516,930	160,870/Braila	260
S-W	690,657	116,099/Arad	200
N-W	182,550	4,665/Cluj	60

**Table 3:** Type 1 PRRSV strains used in this study.

Sequence number	Farm and sequence name	Country	Year	Gene Bank ACC No.	

				*ORF5*	*ORF7*
1	2888	Austria	-	AY875855	-
2	2906/2	Austria	-	AY875862	-
3	2234	Austria	-	AY875858	-
4	AN	Belarus	2001	-	EU071252
5	Bel	Belarus	2004	DQ324669	DQ324699
6	MN	Belarus	1999	-	EU071253
7	Bor	Belarus	2004	-	DQ324701
8	BR	Belarus	2001	-	EU071254
9	Obu	Belarus	2005	DQ324676	DQ324707
10	Okt	Belarus	2004	-	DQ324708
11	PK	Belarus	2002	-	-
12	Sno	Belarus	2004	-	DQ324713
13	Soz	Belarus	2004	DQ324686	DQ324719
14	Soz (2)	Belarus	2006	EU071227	EU071222
15	Soz (3)	Belarus	2006	EU071228	EU071223
16	Vas	Belarus	2005	-	DQ324722
17	MG	Belarus	1999	-	EU071255
18	Vos	Belarus	2004	DQ324690	DQ324725
19	Yus	Belarus	2004	-	DQ324727
20	ZD	Belarus	2000	-	EU071256
21	Zad	Belarus	2004	DQ324694	DQ324729
22	Zap	Belarus	2004	-	DQ324734
23	V-501	Czech Republic	1996	AF253531	-
24	KT	Czech Republic	2006	-	EU071224
25	361–4	Denmark	1994	AY035915	AY035960
26	28639/98	Denmark	1998	AY035912	AY035957
27	2567/96	Italy	1996	AY035932	AY035976
28	2029/97	Italy	1997	AY035930	AY035973
29	IT7	Italy	2004	AY739963	-
30	IT8	Italy	2004	AY739964	-
31	IT15	Italy	2004	AY739971	-
32	IT19	Italy	2002	AY739975	-
33	IT42	Italy	2003	-	-
34	IT62	Italy	2003	AY743932	-
35	Aus	Lithuania	2000	-	AF438362
36	Sid	Lithuania	2000	-	AF438363
37	Che	Poland	2005	-	DQ324703
38	Dob	Poland	2007	-	EU071225
39	Dzi	Poland	2005	-	DQ324705
40	Prz	Poland	2005	-	DQ324711
41	Sok	Poland	2004	-	DQ324715
42	RS	Russia	2005	EU071230	EU071257
43	BK	Russia	2006	EU071231	EU071258
44	BLG	Russia	2006	EU071232	EU071259
45	FR	Russia	2000	-	EU071260
46	GB-1	Russia	1997	-	EU071261
47	GB-2	Russia	2000	-	EU071262
48	RV	Russia	2003	-	EU071263
49	VR	Russia	2005	EU071233	EU071264
50	KM	Russia	1999	-	EU071265
51	KH-1	Russia	2004	-	EU071266
52	KH-2	Russia	2005	EU071234	EU071267
53	KH-3	Russia	2005	EU071235	EU071268
54	SHV	Russia	2006	EU071236	EU071269
55	IN	Russia	2005	EU071237	EU071270
56	KR	Russia	2000	-	EU071271
57	MB-1	Russia	2004	-	EU071272
58	MB-2	Russia	2005	EU071238	EU071273
59	KZ-1	Russia	1999	-	EU071274
60	KZ-2	Russia	2004	EU071239	EU071275
61	DZ	Russia	2000	-	EU071276
62	IL-1	Russia	1997	EU071240	EU071277
63	IL-2	Russia	1999	-	EU071278
64	VD	Russia	1997	-	EU071279
65	NB	Russia	2006	EU071241	EU071280
66	NV-1	Russia	1996	-	EU071281
67	NV-2	Russia	2004	-	EU071282
68	NV-3	Russia	2006	EU071242	EU071283
69	OR	Russia	2000	-	EU071284
70	PMP	Russia	2006	EU071243	EU071285
71	PN	Russia	2000	-	EU071286
72	PR	Russia	1998	-	EU071287
73	SM	Russia	1998	-	EU071288
74	SP	Russia	1999	-	EU071289
75	NE	Russia	1999	-	EU071290
76	SNK-1	Russia	1999	-	EU071291
77	SNK-2	Russia	2004	-	EU071292
78	TM-1	Russia	2000	-	EU071293
79	TM-2	Russia	2005	EU071244	EU071294
80	TT	Russia	1998	-	EU071295
81	TL	Russia	1999	-	EU071296
82	ZV	Russia	2004	EU071245	EU071297
83	UD	Russia	2001	-	EU071298
84	VL-1	Russia	2001	-	EU071299
85	VL-2	Russia	2004	-	EU071300
86	VL-3	Russia	2006	EU071246	EU071301
87	BT-1	Russia	2000	-	EU071302
88	BT-2	Russia	2006	EU071247	EU071303
89	ND-1	Russia	1998	EU071248	EU071304
90	ND-2	Russia	1999	-	EU071305
91	ND-3	Russia	2006	EU071249	EU071306
92	VSH	Russia	2005	EU071250	EU071307
93	GK	Russia	2005	EU071251	EU071308
94	IV3140	South Korea	-	DQ355821	DQ355822
95	CP6874	South Korea	-	EF031042	-
96	1751/93	Spain	1991	AY035935	AY035979
97	CRESA9	Spain	-	DQ009634	-
98	CRESA11	Spain	-	DQ009626	-
99	CRESA13	Spain	-	DQ009637	-
100	CRESA14	Spain	-	DQ009638	-
101	CRESA22	Spain	-	DQ009645	-
102	28/2003	Spain	-	DQ345755	-
103	01RB1	Thailand	2001	-	AY796316
104	Amervac PRRS	Spain	vaccine	-	DQ324698
105	Pyrsvac-183	Spain	vaccine	-	DQ324712
106	Porcilis PRRS	The Netherlands	vaccine	-	DQ324710
107	Porcilis PRRS	Italy	2004	AY743931	-
108	Rom3	Romania	2012	JX075094	-
109	Rom5	Romania	2012	JX075096	-
110	Rom6	Romania	2012	JX075097	-
111	Rom7	Romania	2012	JX090163	-
112	Rom8	Romania	2012	JX090167	-
113	Rom9	Romania	2012	JX090164	-
114	Rom10	Romania	2012	JX090165	-
115	Rom11	Romania	2012	JX090166	-
116	Rom12	Romania	2012	JX099577	-
117	Rom13	Romania	2012	JX099578	-
118	Rom14	Romania	2012	JX099572	-
119	Rom15	Romania	2012	JX099573	-
120	Rom16	Romania	2012	JX099574	-
121	Rom17	Romania	2012	JX099575	-
122	Rom18	Romania	2012	JX099576	-
123	Rom19	Romania	2012	JX099579	-
124	Rom20	Romania	2012	JX105430	-
125	Rom21	Romania	2012	JX105431	-
126	Rom4	Romania	2012	-	JX075095
127	Rom22	Romania	2012	-	JX134062
128	Rom23	Romania	2012	-	JX134063
129	Rom24	Romania	2012	-	JX134064
130	Rom25	Romania	2012	-	JX134065
131	Rom26	Romania	2012	-	JX134066
132	Rom27	Romania	2012	-	JX134067
133	Rom28	Romania	2012	-	JX134068
134	Rom29	Romania	2012	-	JX134069
135	Rom30	Romania	2012	-	JX134070
136	Lelystad	Netherlands	1993	M 96262	M 96262
